# Assessing the quality of chest compressions with a DIY low-cost manikin (LoCoMan) versus a standard manikin: a quasi-experimental study in primary education

**DOI:** 10.1007/s00431-024-05601-8

**Published:** 2024-05-14

**Authors:** Lucía Peixoto-Pino, Santiago Martínez Isasi, Martín Otero Agra, Tina Van Duijn, Javier Rico-Díaz, Antonio Rodriguez Núñez, Roberto Barcala Furelos

**Affiliations:** 1 Faculty of Education Sciences, Universidade de Santiago de Compostela, Santiago de Compostela, Spain; 2 Santiago Martinez-Isasi, Faculty of Nursing, University of Santiago de Compostela, Av. Xoán XXIII, Santiago de Compostela, A Coruña Spain; 3 CLINURSID Research Group, Public Health, Nursing and Medicine Department, Universidade de Santiago de Compostela, PsychiatrySantiago de Compostela, Radiology Spain; 4 Simulation and Intensive Care Unit of Santiago (SICRUS) Research Group, Health Research Institute of Santiago, University Hospital of Santiago de Compostela-CHUS, Santiago de Compostela, Spain; 5 Primary Care Interventions to Prevent Maternal and Child Chronic Diseases of Perinatal and Developmental Origin (RICORS), Instituto de Salud Carlos III, RD21/0012/0025 Madrid, Spain; 6 School of Nursing From Pontevedra, Universidade de Vigo, Pontevedra, Spain; 7 REMOSS Research Group, Faculty of Education and Sport Sciences, University of Vigo, Pontevedra, Spain; 8 Human Performance Research Center, University of Technology Sydney, Sydney, Australia; 9 Swiss Lifesaving Society, Sursee, Switzerland; 10 School of Physical Education, Sport and Exercise Science, University of Otago, Dunedin, New Zealand; 11 Paediatric Critical, Intermediate and Palliative Care Section, Santiago de Compostela’s University Hospital, Santiago de Compostela, Spain; 12 ESCULCA Knowledge and Educational Action Research Group, Universidade de Santiago de Compostela, A Coruña, Spain

**Keywords:** Hands-only cardiopulmonary resuscitation, Low-cost, Schoolchildren, Chest compressions, Educational program

## Abstract

Extending the access to cardiopulmonary resuscitation (CPR) training to a wider public is an important step in increasing survivability of out-of-hospital cardiac arrest. However, often price and maintenance of CPR manikins are barriers that prevent training at schools. This study aims to evaluate the learning of hands-only (HO) CPR by practicing with a low-cost manikin (LoCoMan) with visual qualitative feedback and to compare the results with the skills acquired by practice on a conventional manikin. A quasi-experimental study with 193 schoolchildren (10 to 12 years old) who were allocated to two groups: the LoCoMan group was taught via an integrative approach (science combined with physical education (PE)) and practiced on a handmade manikin, and a control group practiced in a traditional setting with a commercial manikin (Resusci Junior, Laerdal, Norway). All participants practiced for 1 hands-on skill session before performing a post-test on an instrumented CPR manikin. The outcomes including HO-CPR performance variables were compared between groups. The LoCoMan and control groups both achieved acceptable percentage of HO-CPR quality (57% and 71%, *p* = 0.004). Among 6th-graders, there were no significant differences in HO-CPR quality between LoCoMan 68% and control 71%, *p* = 0.66. The control group achieved better chest compression depth while the LoCoMan group showed more compressions with adequate chest recoil.

*     Conclusion*: Schoolchildren are able to build and use a low-cost manikin with visual feedback. The integrative learning approach used in this study may be a feasible alternative methodology for training and learning HO-CPR in schools when commercial manikins are not available.

**Table:** 

**What is Known:** *• Access to CPR training should be universal and independent of age, location, financial means, or access to qualified instructors.* *• Scientific societies promote the implementation of CPR in schools, so that teachers and schoolchildren can play a multiplier role in their environment, but the gap in CPR learning is related to cultural, economic factors or access to resources and materials.*	
**What is New:** *• LoCoMan may be a useful device for teaching and learning CPR in schoolchildren from the age of 10 and upwards.* *• LOCOMAN shows that it is feasible and possible to build a low-cost manikin (about €5 in the European Region) and to integrate it into an integrative educational project, and outlines how this could be done. this approach can be an incentive for teachers to attempt teaching CPR, but also for education outside the formal environment.*

**Supplementary Information:**

The online version contains supplementary material available at 10.1007/s00431-024-05601-8.

## Introduction

It is commonly accepted that early training in cardiopulmonary resuscitation (CPR) increases the likelihood that bystanders can intervene in an emergency and eventually improve the outcomes of out-of-hospital cardiac arrest (OHCA) [1, 2]. Access to CPR training should be universal and independent of age, location, financial means, or access to qualified instructors. To expand the reach of CPR training, the European Resuscitation Council (ERC) and the American Heart Association (AHA) encourage widespread CPR education [3, 4] and the declaration “KIDS SAVE LIVES,” supported by the World Health Organization (WHO), promotes the implementation of CPR at schools so that teachers and schoolchildren can play a multiplying role in their environment [5]. However, there is still a gap in CPR learning related to several factors, due to cultural and economic factors or access to resources and materials. To try to overcome these barriers, low-cost manikins can be an option for mass CPR teaching and training [6]. Currently, there are various low-cost devices that may be used as an alternative to conventional manikins; some low-cost devices are prefabricated, such as for example an auditory feedback heart made of plastic (5), a natural rubber manikin (6), or a children’s pillow [7–9].

More recently, a do-it-yourself (DIY) movement has emerged [10, 11] which is based on the construction and self-development of devices that can simulate a human torso or be useful for teaching resuscitation maneuvers, in order to promote mass training at low cost. Most DIY models we are aware of are either made with conventional materials, such as toilet paper, T-shirts, or towels [10, 12, 13], or based on recycled material, especially plastic bottles [6, 14]. However, there is still a lack of knowledge on the usability of such manikins in the school. Studies on the acquisition of CPR skills via DIY manikin compared to commercial manikins are scarce. The development of DIY manikins must evolve and diversify to include models that are suitable for different contexts, learning outcomes, and target groups. In a pedagogically ideal case, a DIY manikin may be able to replace with ingenuity those elements that have been shown to be effective in teaching resuscitation—such as feedback [15, 16], motivation [17], cognitive involvement [7], or gamification [18]—and evaluate their effect on the acquisition of skills.

Traditionally, conventional and unconventional manikins have focused on achieving the proper depth or rate of compressions, but it is well known that a key factor in resuscitation is its continuity, i.e., avoiding interruption of chest compressions (CC) [19], especially when performing the hands-only CPR (HO-CPR) technique [20]. However, to our knowledge, there is no low-cost manikin for lay people that helps to train and understand this physiological concept.

Therefore, this study aims to evaluate the learning of CC skills by practicing with a low-cost manikin (LoCoMan) with visual qualitative feedback for continuous CC and to compare the results with the skills acquired by practice on a conventional manikin.

## Material and method

### Participants

A convenience sample of 193 schoolchildren from Spain participated in this research, aged between 10 and 12 years, corresponding to two academic years (5th and 6th year of primary school). The data broken down by groups and sex are shown in Table 1. Exclusion criteria were any physical or mental impairments and any previous experience (theoretical or practical) in cardiopulmonary resuscitation.

**Table 1:** Characteristics of the sample

Variables		Total *n* = 193		LoCoMan group *n* = 91		Control group *n* = 102		*p* value
Academic year		*n*	(%)	*n*	(%)	*n*	(%)	
5ºPE [10–11 years]		106	(55%)	45	(50%)	61	(60%)	*p* = 0.15
6ºPE [11–12 years]		87	(45%)	46	(51%)	41	(40%)	
Sex		*n*	(%)	*n*	(%)	*n*	(%)	
Female		106	(55%)	56	(61%)	50	(49%)	*p* = 0.08
Male		87	(45%)	35	(39%)	52	(51%)	

*PE* primary education

The research was approved by the Ethics Committee of the Faculty of Education and Sport Sciences–University of Vigo (Spain) that supported (Code: 03–250322). The study was authorized by the educational department of the participating schools and the parents (via written informed consent).

### Study design

**Fig. 1:**
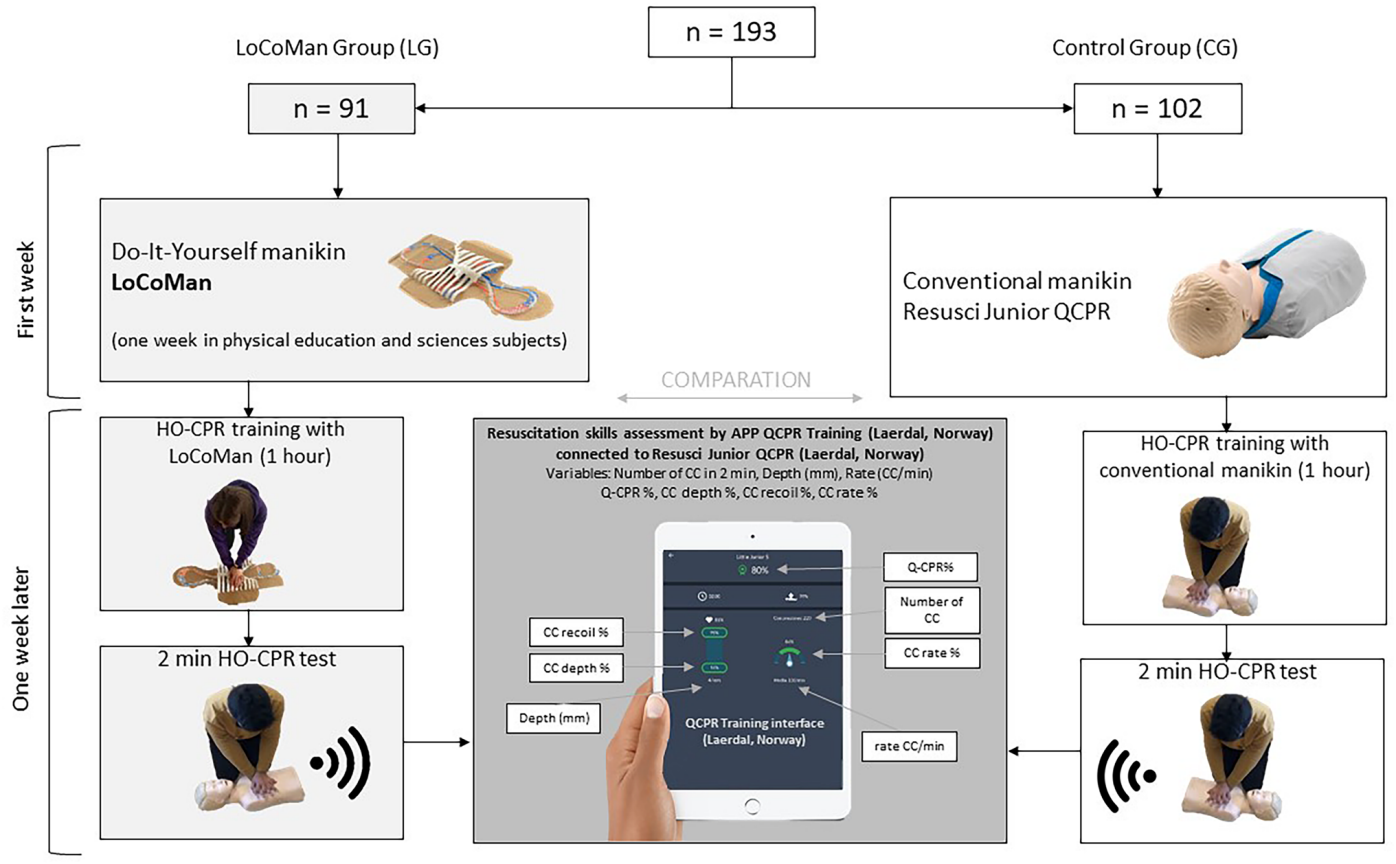
Flowchart design

A quasi-experimental and cross-sectional study was carried out using two nonrandomized intervention groups; the LoCoMan group (LG) trained with a hand-made low-cost manikin and a control group (CG) practiced with a conventional pediatric manikin model, Resusci Junior (Laerdal, Norway) (Fig. 1).

### LoCoMan: educational project principles

**Fig. 2:**
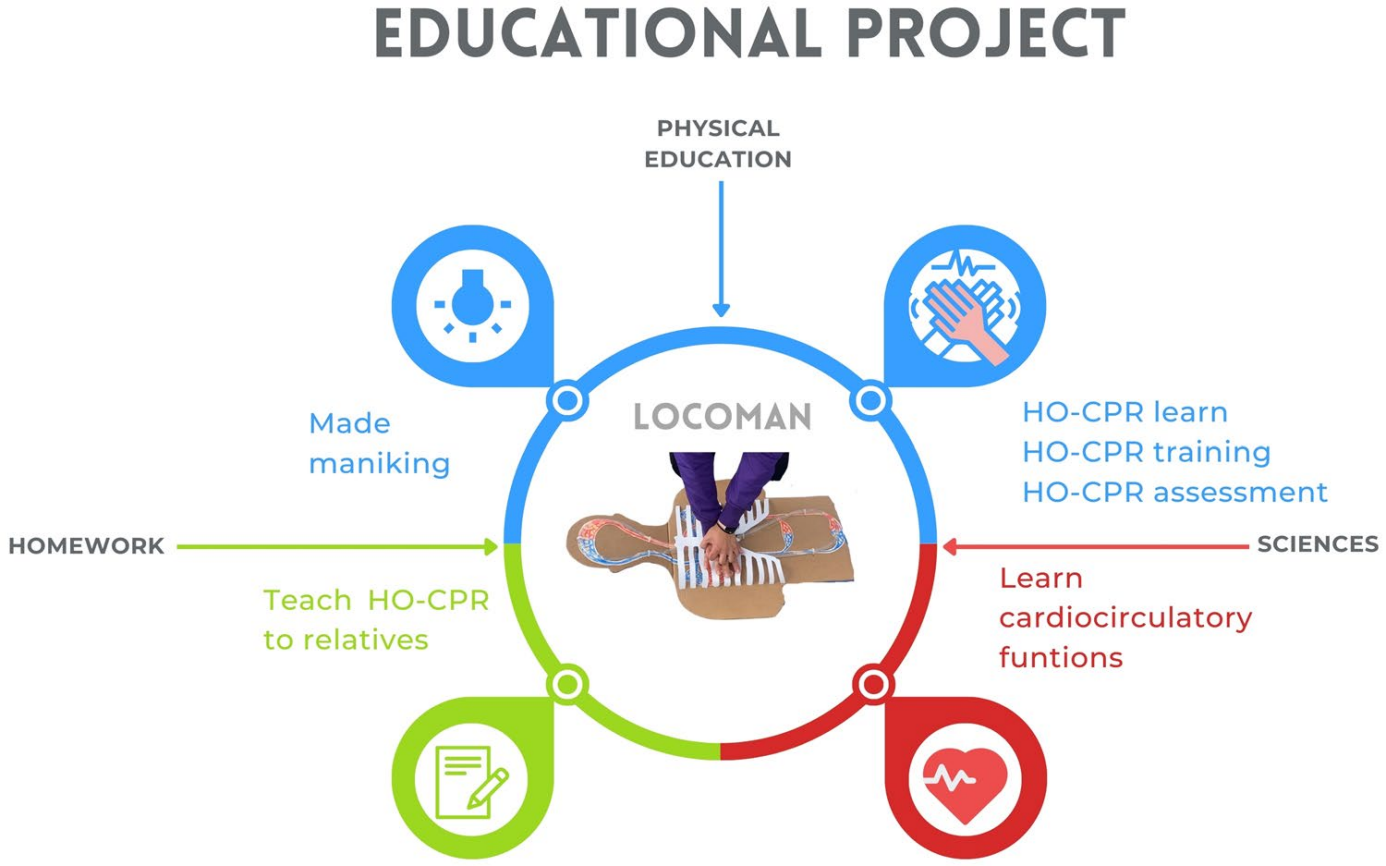
Integrative educational project learn-by-doing LoCoMan

This project was based on the principles of significant, transversal, and integrative learning [21, 22], focused on understanding the process by which continuous chest compressions achieve continuous circulation and cerebral perfusion. The LG built their manikins during one academic week in the physical education subject (2 sessions), in a coordinated manner with the subject of science (2 sessions), during which cardiocirculatory functions were learned (Fig. 2). Children in the CG received the same science lesson, but without a comprehensive approach (no connection between contents).

**Fig. 3:**
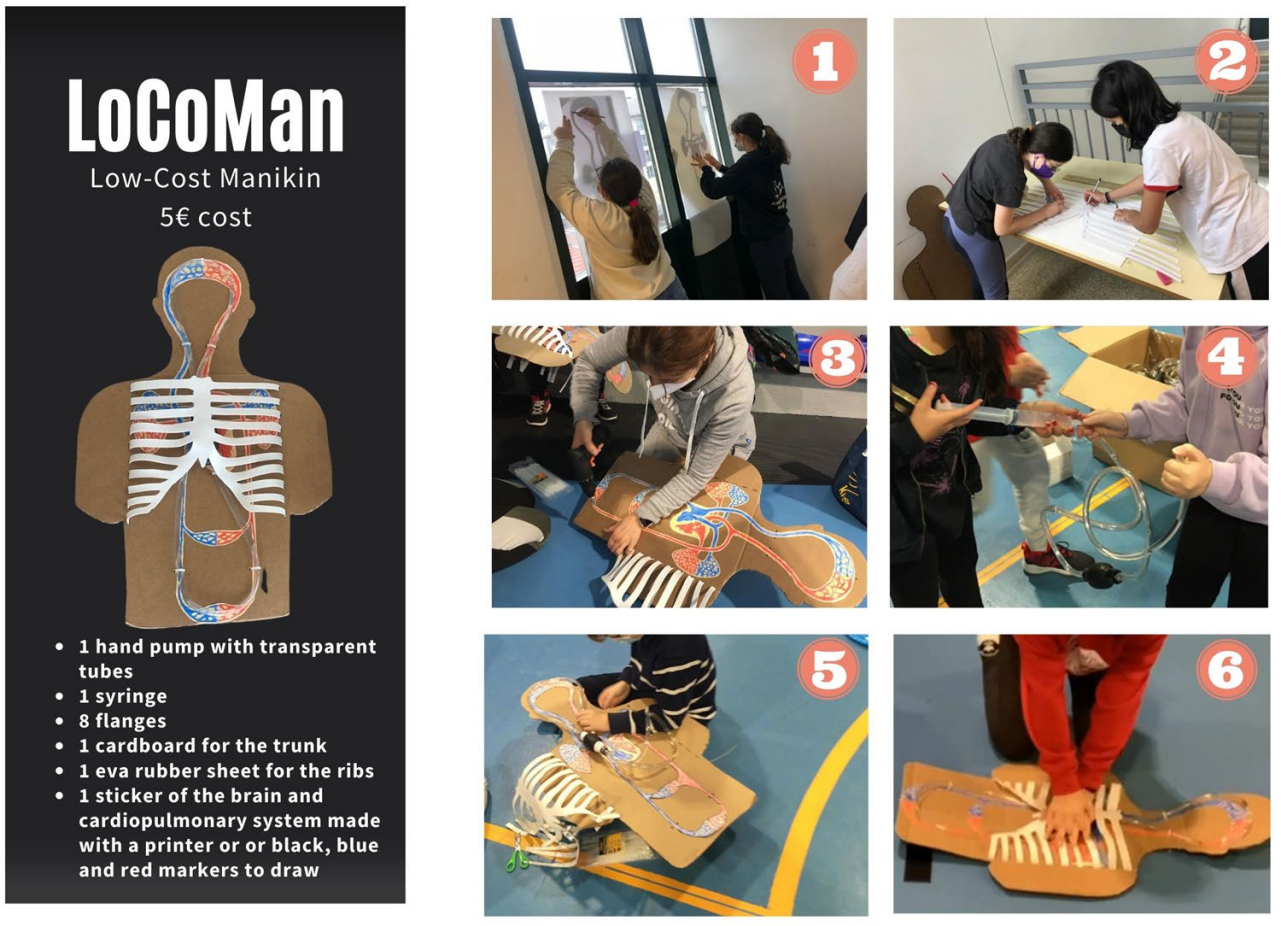
How to make a LoCoMan (step by step) and in a supplementary video online

- Science subject: participants were introduced to the cardiocirculatory system and the effect of HO-CPR on circulation. At the same time, they studied the functions of blood and the beating of the heart, the cardiac cycle, the transport of blood, and the need for oxygen in different organs such as the brain.
- Physical education subject: participants built their manikin under guidance from a teacher. In small groups, they helped each other in the different phases of the build, which provoked collaboration and reflection. The materials and instructions for the build of LoCoMan are described in Fig. 3; more detailed instructions are available in an online supplementary video and assembly manual.
- Homework: in a first step before building LoCoMan, inspired by the flipped or inverted classroom theory [23, 24], the schoolchildren introduced the project to their families, and together they had to get some of the materials, such as cardboard to draw the human silhouette. In this way, the students began to integrate human anatomy, cardiac physiology, and cardiopulmonary resuscitation. In a second step, at the end of the learning unit, schoolchildren had to take LoCoMan to their homes and teach HO-CPR to their relatives.

### LoCoMan’s feedback mechanism (Fig. 3)

LoCoMan’s blood circuit is made up of transparent plastic tubes, into which water (and some bubbles) had previously been introduced with a simple syringe. Once the “circulation tubes” are sealed, the water and numerous air bubbles are visible. This visually simulates the transport of oxygen by the blood. Visual feedback is based on visualizing the flow of fluid that is percussed (simulating the volume of systolic ejection) with each CC. The movement of the fluid, its circulation and the irrigation of the brain, is easily perceptible during the maneuvers, as well as the immediate blood flow interruption when CC are interrupted (see supplementary video).

### CPR training (LG and CG)

CPR training was guided by a physical education teacher, who had previously been trained by accredited instructors [25]. During a 1 h-long physical education class, the following phases were carried out:

1. An explanation of the chain of survival was provided, including the sequence: recognizing cardiac arrest, calling 112, performing HO-CPR, and following the demonstration of chest compressions: depth, rate, and correct position of execution. In the LG, feedback was provided by the movement of air bubbles through the “blood circuit” during compression, which should not stop. In the CG, feedback was provided by a tablet connected to the Resusci Junior (Laerdal, Norway) through the APP QCPR Training for IOS (Laerdal, Norway). The students in this group were instructed how to interpret the feedback from the tablet regarding three indicators: depth, full chest recoil, and rhythm (rate).
2. Practical demonstration of how to perform CPR on the manikin (the same for LG and CG).
3. The students performed CC guided by the teacher (the same for LG and CG).
4. Schoolchildren performed HO-CPR to the rate of the “La Macarena” song [26] (the same for LG and CG).
5. Schoolchildren performed HO-CPR without any kind of teacher feedback (the same for LG and CG).

### Assessment of skills and analysis variables

HO-CPR performance was analyzed during a separate testing session after the last practice session. Each student performed an individual test of 2 min. The assessment was carried out using the Little Junior QCPR manikin (Laerdal, Norway) programmed under the European Resuscitation Council guidelines for resuscitation 2021 (ERC2021): The gold standard describes chest compression (CC) depth of 50–60 mm, full chest recoil, and a CC rate between 100 and 120 compressions per minute. Using the QCPR instructor app (Laerdal, Norway), the following variables were obtained: depth, full chest recoil, and CC rate, as well as the global value of the quality of HO-CPR (QHO-CPR) in percentage. This device is widely used in the scientific literature for the evaluation of skills during resuscitation in simulation studies [18, 27, 28].

Manikin, interface, and variables can be seen in Fig. 1. The analysis variables were (a) number of CC in 2 min, (b) mean rate (*R*) of CC per minute during 2 min, (c) average depth of CC in mm (*D*) and as percentage variables (d) overall QHO-CPR, (e) CC with adequate chest recoil, (f) CC with adequate depth, and (g) CC with adequate rate.

The percentage of students who reached different percentages of QHO-CPR was analyzed for 4 intervals: 0–24%, 25–49%, 50–69% and ≥ 70%. A result equal to or greater than 70% has been established as the highest standard [29].

### Statistical analysis

Based on our hypothesis, CG and LG were compared in each dependent variable. An additional round of analyses was conducted within each school year (5th graders and 6th graders). All analyses were performed with the IBM SPSS Statistics Software version 20 for Windows. To describe the quantitative variables, measures of central tendency (median) and dispersion (interquartile range, IQR) were used. After checking the normality of the distributions with the Kolmogorov–Smirnov test, group (CG vs. LG) comparisons of the continuous variables were made with the Mann–Whitney *U* test (nonparametric test) or with Student’s *t* test (parametric test). In statistically significant comparisons, the effect size (ES) was calculated using Rosenthal’s *r* test (nonparametric test) or Cohen’s *d* test (parametric test). To define the ES, the following classification was used: < 0.2 trivial; 0.2–0.5 small; 0.5–0.8 moderate; 0.8–1.3 large; ≥ 1.3 very large. For the description of the percentage variables, absolute frequencies and relative frequencies were used. For the comparison of the groups in the percentage variables, the chi square test was used. In statistically significant comparisons, the ES was calculated using Cramer’s *V* test. To define the ES, the following classification was used: 0.1–0.3 small; 0.3–0.5 medium; ≥ 0.5 large. In the case of multiple comparisons between the groups (chi square), a *p* value of 0.012 (0.05/4) was used for the Bonferroni correction. A significance level of *p* = 0.05 was assigned for all other analyses.

## Results

Table 2 shows the dependent variables. The median number of CC performed during 2 min by the LG was 196 [161–221] while in the CG it was 217 [195–235], *p* = 0.001. In the disaggregated analysis of continuous variables, mean rate and mean depth also present significant differences, rate: LG 99 [87–109] vs. CG 109 [100–122], *p* < 0.001 y, depth: LG 43 mm [38–49] vs. CG 49 mm [43–55], *p* < 0.001, in the latter case with a large effect size (0.70).

**Table 2:** CPR variables

Variable	LoCoMan group No. = 91		Control group No. = 102		*p* values (*p*) and effect size (ES)
	Median	IQR	Median	IQR	
Number of CC	196	(161–221)	217	(195–235)	*p* = 0.001 (ES 0.26)
Mean rate (CC/min)	99	(87–109)	109	(100–122)	*p* < 0.001 (ES 0.34)
Mean depth (mm)*	43	(38–49)	49	(43–55)	*p* < 0.001 (ES 0.70)
QHO-CPR (%)	57	(18–84)	71	(42–86)	*p* = 0.04 (ES 0.15)
CC with full chest recoil (%)	100	(92–100)	98	(74–100)	*p* = 0.01 (ES 0.18)
CC with adequate depth (%)	3	(0–47)	40	(5–82)	*p* < 0.001 (ES 0.33)
CC with adequate rate (%)	31	(13–64)	38	(21–62)	*p* = 0.30

IQR interquartile range, *N* absolute frequency, *(%)* relative frequency

For quantitative variables: Mann–Whitney’s *U* test with Rosenthal’s test for effect size

For effect size classification: < 0.2: trivial; 0.2–0.5: small; 0.5–0.8: moderate; 0.8–1.3: large; > 1.3: very large

For qualitative variables: chi square test with Cramer’s *V* test for effect size

For effect size (ES) classification: 0.1–0.3: small; 0.3–0.5: medium; ≥ 0.5: large

^*^For quantitative variables: Student’s *t* test with Cohen’s *d* test for effect size

In the analysis of the percentage of quality, LG performed at 57% [18–84] vs. CG 71% [42–86], *p* = 0.04, with a small effect size (0.15). The chest recoil rate was LG 100% [92–100] vs. CG 98% [74–100], *p* = 0.01; the percentage of adequate depth was LG 3%[0–47] vs. CG 40%[5–82], *p* < 0.001; and the percentage of CC at an adequate rate was not significantly different between groups: LG 31% [13–64] vs. CG 38% [21–62], *p* = 0.24.

In the analysis of the percentage of QHO-CPR in the 5 sectors [0–24%, 25–49%, 50–69%, and ≥ 70%], at a global level, no significant differences are found in the group of children who reach the highest sector of ≥ 70%.

**Fig. 4:**
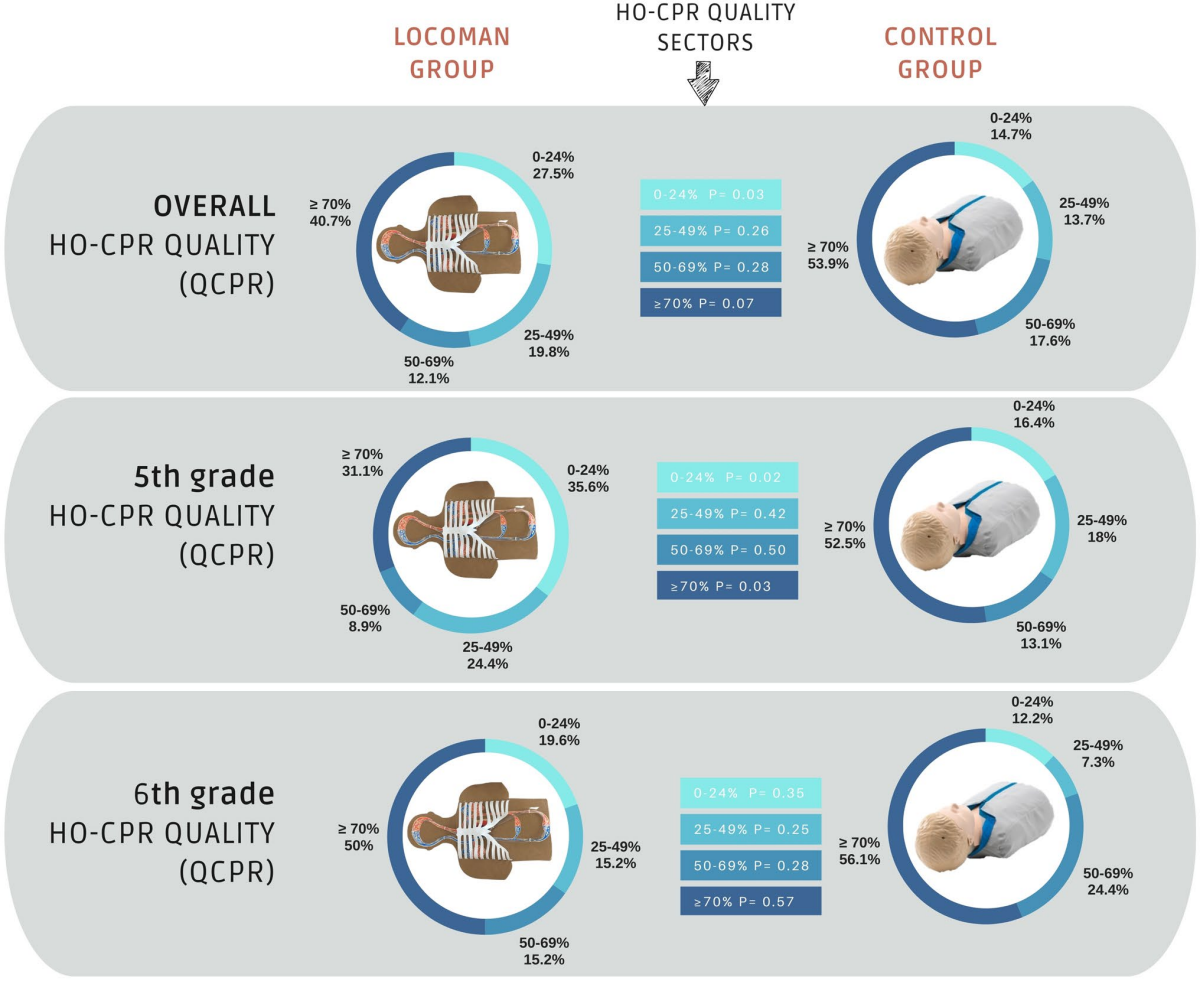
Quality CPR sectors and percentage of schoolchildren by group and grade

In the 5th grade, the best percentages are obtained by the control group, but in the 6th grade, the values of the CG and LG groups are similar, without significant differences in any sector (Fig. 4).

In the analysis by school grades (supplementary Table 1), in the cohort of 5th grade of primary education, all the variables showed significant differences in favor of the CG except in CC with adequate chest recoil. In the global analysis of quality (QHO-CPR variable), the CG (71 [72–89]) performed better than the LG (39 [4–82], *p* < 0.02). Analyzing the data from the 6th grade cohort of primary education, there were significant differences between groups in the percentage variables, but no significant differences in the QHO-CPR.

## Discussion

The aim of this study was to evaluate the learning of CC skills by practicing with a DIY manikin for continuous CC and to compare the results with the skills acquired by practice on a conventional manikin. Our findings corroborate the hypothesis that LoCoMan may be a useful device for teaching and learning HO-CPR in schoolchildren from the age of 10 and upwards.

We decided to work with 10-year-old schoolchildren, since chest compressions can be learned at this age with adequate training [30]. However, neither the group trained with LoCoMan nor the group that used the conventional manikin reached the recommended depth of compression (50–60 mm) [31]. These findings may be related to the relatively low body mass at this age: the relationship of body mass and achieved depth of compressions is a limiting factor for children’s success at learning this skill [32, 33]. It should also be noted that the LoCoMan model cannot be compressed to the full depth of 50 mm (maximal depth is 35 mm). This is a probable explanation for the finding that the control group obtained better results in depth variable in this study. Part of the purpose of building LoCoMan was to provide feedback on the continuity of compressions for continuous cerebral perfusion, a fundamental concept in HO-CPR. Based on the results obtained from number of CC, rate, and mean rate percentage, we were able to verify that there was no interruption, neither in the control group using electronic feedback, nor in the LoCoMan group.

In the cohort analysis (i.e., separated by school year), LoCoMan led to better CPR quality in 6th graders (aged 11 and 12). Indeed, they performed practically equal to the control group, approaching the gold standard of 70% [29]. In other CPR variables such as full chest recoil or CC rate, significant variations were found between the two models, but these differences would not have clinical relevance because their values are close or very close to the gold standard of the resuscitation guidelines [34] (i.e., rate with LoCoMan was 104 CC/min vs. Laerdal Manikin 118 CC/min, gold standard 100–120 CC/min).

Hand-made manikins may contribute to the improvement of individual CPR and can make mass collective teaching possible, especially in low-resource settings [6] when there is no industry or government support. We consider that this project may be relevant because it could help promote the teaching of basic life support in schools or in informal contexts[6–10, 14, 35], which might have a significant impact on bystanders who perform CPR [4], for example, by increasing confidence and readiness to act in an emergency [17, 36].

A relevant feature of LoCoMan is the qualitative feedback that allows the schoolchild to observe how the simulated blood circuit flows during chest compressions. To our knowledge, this is the only model that has visual feedback and integrates knowledge of circulatory physiology. Feedback during practice has been associated with better CPR quality and more effective learning [15], helping students achieve mastery of skills and shortening demonstration time [10]. Another noteworthy aspect is that throughout the training process, no manikin suffered significant deterioration, so this pilot tool is potentially sustainable for training or learning, and although it is not commercialized, it can be locally manufactured since its components are readily available.

Most OHCAs occur at home (26). One additional aim of this educational project was to enable families to practice CPR at their homes. For this reason, the intervention included several “at-home steps”: Before building their manikin, learners involved their care-takers and relatives in the collection of recyclable materials to bring to school, and after the manikin was finished, the schoolchildren had to teach the skills acquired to their relatives. This process was not analyzed as part of this paper, but it is also a mission of the project that should be evaluated in the future.

The presented project conforms to the main principles of KIDS SAVE LIVES: the dissemination of knowledge and training, encouraging fun and excitement during CPR (3). From an educational perspective, learning is stronger and more durable when it is based on connection of content, rather than analytical, isolated, or mechanical learning. New learning methodologies and materials emerge and promote learning-by-doing (5). LoCoMan is a project that combines the subject of science (knowledge of the human body) and physical education (first aid), all the while keeping the learner at the center, as the protagonist promoting their own learning progress [21, 22].

This work has important practical implications: it shows that it is feasible and possible to build a low-cost manikin (about €5 in the European Region) and to integrate it into an integrative educational project and outlines how this could be done. As a general reference, the cost of 100 LoCoMan units is less than 2 commercial manikins with feedback (i.e., as used in this study), and the learning outcomes are comparable. These and other evidence-based alternatives to expensive manikin training may be an incentive for teachers to attempt teaching CPR, but also for education outside the formal environment.

### Study limitations

We acknowledge that this study has a few limitations. The sample was located in a specific region, so it is not representative of all children worldwide. As the classes were grouped by academic years, there was a slight age overlap around the age of 11 years. A distribution by biological age (10, 11, and 12 years) could be more appropriate, but due to the educational dynamics of the school system, this has not been possible. Another factor that is necessary to note is that the LoCoMan is part of a multidisciplinary educational project, and this circumstance might improve the motivation of schoolchildren and relatives to perform well. A relevant and limiting aspect of the study is that a portion of the learning in the LoCoMan group may possibly be influenced by the overall project rather than exclusive training in HO-CPR. This methodological limitation also serves as a strength of this project, which extends beyond the building of a manikin. Another limitation is that, as each student made their own LoCoMan, there may have been small variations between manikins as it is not a standardized model. The LoCoMan structure allows a maximum compression depth of 35 mm (based on measurements with a CPR meter) (Laerdal, Norway, see supplementary video online) which may be the most plausible justification for achieving 3% of compressions at the indicated depth compared to the 40% achieved by the children who trained on the commercial manikin. Future versions of LoCoMan will address this issue.

## Conclusion

The use of a low-cost, hand-made manikin with visual feedback might be an alternative for training and learning CPR in schools, especially when commercial manikins are not available. Low-cost manikins can be integrated into an educational project that promotes learning of CPR.

### Supplementary Information

Below is the link to the electronic supplementary material.

Supplementary file1 (MP4 26072 KB)

Supplementary file2 (DOCX 16 KB)

Supplementary file3 (DOCX 65 KB)

## Abbreviations

AHA: American Heart Association
CC: Chest compressions
CPR: Cardiopulmonary resuscitation
CG: Control group
DIY: Do-it-yourself
ERC: European Resuscitation Council
ES: Effect size
HO-CPR: Hands-only CPR (continuous chest compression)
IQR: Interquartile range
LoCoMan: Low-cost manikins
LG: LoCoMan group
OHCA: Outcomes of out-of-hospital cardiac arrest
PE: Physical education
QHO-CPR: Quality of HO-CPR in percentage (0–100%)
