# Supplementary material for: Assessing the quality of chest compressions with a DIY low-cost manikin (LoCoMan) versus a standard manikin: a quasi-experimental study in primary education
Source: Eur J Pediatr. 2024 May 14;183(8):3337–46. doi: 10.1007/s00431-024-05601-8 (PMC11263256; doi:10.1007/s00431-024-05601-8)
Supplement: Supplementary file 2 — Supplementary file2 (DOCX 16 KB) [file 431_2024_5601_MOESM2_ESM.docx]

| supplementary table 1: CPR variables by age | | | | | |
| --- | --- | --- | --- | --- | --- |
| 5 th PE [10 and 11 yo] | | | | | |
| variables | Locoman group (LG)  No. = 45 | | Control group (CG)  No. = 61 | | Significance |
|  | Median | IQR | Median | IQR |  |
| Number of CC | 189 | (138 - 204) | 207 | (190 - 223) | p = 0.001 (0.32) |
| Rate (CC/min) | 96 | (86 - 103) | 106 | (97 - 116) | p < 0.001 (0.36) |
| Depth (mm) | 41 | (37 - 48) | 49 | (42 - 53) | p < 0.001 (0.40) |
| CPR Quality (%) | 39 | (4 - 82) | 72 | (38 - 89) | p = 0.02 (0.24) |
| CC with adequate recoil (%) | 100 | (98 - 100) | 99 | (74 - 100) | p < 0.002 (0.30) |
| CC with adequate depth (%) | 2 | (0 - 36) | 40 | (5 - 77) | p < 0.001 (0.37) |
| CC with adequate rate (%) | 28 | (9 - 46) | 43 | (24 - 67) | p = 0.007 (0.26) |
| 6 th PE [11 and 12 yo] | | | | | |
| variables | Locoman group (LG)  No. = 46 | | Control group (CG)  No. = 41 | | Significance |
|  | Median | IQR | Median | IQR |  |
| NCC | 215 | (169 - 224) | 233 | (211 - 249) | p = 0.008 (0.29) |
| R (CC/min) * | 104 | (89 - 110) | 118 | (103 - 127) | p < 0.001 (0.84) |
| D (mm) | 46 | (40 - 51) | 49 | (44 - 57) | p = 0.011 (0.27) |
| QHO-CPR (%) | 68 | (31 - 88) | 71 | (54 - 81) | p = 0.66 |
| CC with full chest recoil (%) | 98 | (82 - 100) | 93 | (72 - 100) | p = 0.28 |
| CC with adequate depth (%) | 13 | (0 - 59) | 33 | (6 - 91) | p = 0.005 (0.30) |
| CC with adequate rate (%) | 42 | (18 - 80) | 32 | (13 - 55) | p = 0.23 |
| NCC: Number of CC. A: Mean rate (CC/min). D: Mean depth (mm), QHO-CPR: Overall quality of CPR in %. CC: Chest compression. PE. Primary school  IQR: Interquartile range; N: Absolute frequency; (%): Relative frequency.  For quantitative variables: Mann-Whitney's U test with Rosenthal's test for Effect Size  * For quantitative variables: Student's t test with Cohen's d test for Effect Size  For Effect Size classification: < 0.2: Trivial; 0.2 - 0.5: Small; 0.5 - 0.8: Moderate; 0.8 - 1.3: Large; > 1.3: Very Large  For Effect Size classification: 0.1 - 0.3: Small; 0.3 - 0.5: Medium; ≥0.5:Large | | | | | |
