# Supplementary material for: Assessing the quality of chest compressions with a DIY low-cost manikin (LoCoMan) versus a standard manikin: a quasi-experimental study in primary education
Source: Eur J Pediatr. 2024 May 14;183(8):3337–46. doi: 10.1007/s00431-024-05601-8 (PMC11263256; doi:10.1007/s00431-024-05601-8)
Supplement: Supplementary file 3 — Supplementary file3 (DOCX 65 KB) [file 431_2024_5601_MOESM3_ESM.docx]

**Title**

Assessing the Quality of Chest Compressions with a DIY Low-Cost Manikin (LOCOMAN) versus a Standard Manikin: A Quasi-Experimental Study in Primary Education

**Authors**

Lucía Peixoto-Pino^a^. MEd., ^*^Santiago Martínez Isasi^b,c,d,e^. Ph. D., Martín Otero Agra^f,g^ . Ph. D

Tina Van Duijn^h,i,j^ Ph. D., Javier Rico-Díaz^a^ Ph. D., Antonio Rodriguez Núñez^b,c,d,e,k^. Ph. D^.^

Roberto Barcala Furelos ^d,e,g^. Ph. D.

^a^ Faculty of Education Sciences.Universidade de Santiago de Compostela, Santiago de Compostela, Spain

^b^ Faculty of Nursing. University of Santiago de Compostela, Santiago de Compostela, Spain

^c^ CLINURSID Research Group, Psychiatry, Radiology, Public Health, Nursing and Medicine Department, Universidade de Santiago de Compostela, Santiago de Compostela, Spain

^d^ Simulation and Intensive Care Unit of Santiago (SICRUS) Research Group, Health Research Institute of Santiago, University Hospital of Santiago de Compostela-CHUS, Santiago de Compostela, Spain

^e^ Primary Care Interventions to Prevent Maternal and Child Chronic Diseases of Perinatal and Developmental Origin (RICORS), RD21/0012/0025, Instituto de Salud Carlos III, Madrid, Spain

^f^ School of Nursing from Pontevedra, Universidade de Vigo, Pontevedra, Spain

^g^ REMOSS Research Group. Faculty of Education and Sport Sciences, University of Vigo, Pontevedra, Spain

^h^ Human performance research center, University of Technology Sydney, Australia

^i^ Swiss Lifesaving Society, Switzerland

^j^ School of Physical Education, Sport and Exercise Science, University of Otago, New Zealand

^k^ Paediatric Critical, Intermediate and Palliative Care Section, Santiago de Compostela’s University Hospital, Santiago de Compostela, Spain

***Corresponding author.**

Santiago Martinez-Isasi; Faculty of Nursing. University of Santiago de Compostela, Av. Xoán XXIII, Spain. +34 881811250. Email: [santiago.martinez.isasi@usc.es](mailto:santiago.martinez.isasi@usc.es)

**Keywords:** Hands-only cardio-pulmonary resuscitation, low-cost, Schoolchildren, Chest compressions, Educational program

**Funding**

Research funded by Instituto de Salud Carlos III (ISCIII) -PI20/01355- Co-funded by the European Union (EU).

Research funded by Instituto de Salud Carlos III (ISCIII) -PI23/00687- Co-funded by the European Union (EU).

**Conflict of Interest Disclosures**

None reported.

**ABSTRACT**

Purpose

Extending the access to cardiopulmonary resuscitation (CPR) training to a wider public is an important step in increasing survivability of out-of-hospital cardiac arrest. However, often price and maintenance of CPR manikins are barriers that prevent training at schools.

This study aims to evaluate the learning of Hands Only (HO) CPR by practicing with a Low Cost Manikin (LoCoMan) with visual qualitative feedback and to compare the results with the skills acquired by practice on a conventional manikin

Methods

A quasi-experimental study with 193 schoolchildren (10 to 12 years old) who were allocated to two groups: LoCoMan group was taught via an integrative approach (science combined with Physical Education (PE)) and practiced on a handmade manikin, and a control group practiced in a traditional setting with a commercial manikin (Resusci Junior, Laerdal, Norway). All participants practiced for 1 hands-on skill session before performing a post-test on an instrumented CPR manikin. The outcomes including HO-CPR performance variables were compared between groups.

Results

LoCoMan and control group both achieved acceptable percentage of HO-CPR quality (57% and 71%, p = 0.004). Among 6th-graders, there were no significant differences in HO-CPR quality between LoCoMan 68% vs. control 71%, p = 0.66. The control group achieved better chest compression depth while the LoCoMan group showed more compressions with adequate chest recoil.

Conclusions.

Schoolchildren are able to build and use a low-cost manikin with visual feedback. The integrative learning approach used in this study may be a feasible alternative methodology for training and learning HO-CPR in schools when commercial manikins are not available.

**ABBREVIATIOSN AND ACRONYMS**

AHA: American Heart Association

CC: chest compressions

CPR: cardiopulmonary Resuscitation

CG: control group

DIY: Do-It-Yourself

ERC: European Resuscitation Council

ES: Effect size

HO-CPR: Hands Only CPR (continuous chest compression)

IQR: interquartile range,

LoCoMan: Low-cost manikins

LG: LoCoMan group

OHCA: outcomes of out-of-hospital cardiac arrest

PE: Physical Education

QHO-CPR: quality of HO-CPR in percentage (0-100%)

**What is known**

Access to CPR training should be universal and independent of age, location, financial means, or access to qualified instructors.

Scientific societies promote the implementation of CPR in schools, so that teachers and schoolchildren can play a multiplier role in their environment, but the gap in CPR learning is related to cultural, economic factors or access to resources and materials

**What is new**

LoCoMan may be a useful device for teaching and learning CPR in schoolchildren from the age of 10 and upwards.

LOCOMAN shows that it is feasible and possible to build a low-cost manikin (about €5 in the European Region) and to integrate it into an integrative educational project, and outlines how this could be done. this approach can be an incentive for teachers to attempt teaching CPR, but also for education outside the formal environment.

**INTRODUCTION**

It is commonly accepted that early training in Cardiopulmonary Resuscitation (CPR) increases the likelihood that bystanders can intervene in an emergency and eventually improve the outcomes of out-of-hospital cardiac arrest (OHCA) [1, 2]. Access to CPR training should be universal and independent of age, location, financial means, or access to qualified instructors. To expand the reach of CPR training, the European Resuscitation Council (ERC) and the American Heart Association (AHA) encourage widespread CPR education [3,4] and the declaration "KIDS SAVE LIVES”, supported by the World Health Organization (WHO), promotes the implementation of CPR at schools, so that teachers and schoolchildren can play a multiplying role in their environment [5]. However, there is still a gap in CPR learning related to several factors, due to cultural, economic or access to resources and materials. To try to overcome these barriers, low-cost manikins can be an option for mass CPR teaching and training [6]. Currently there are various low-cost devices that may be used as an alternative to conventional manikins; some low-cost devices are prefabricated, such as for example an auditory feedback heart made of plastic (5), a natural rubber manikin (6) or a children´s pillow [7–9].

More recently, a DO-IT-YOURSELF (DIY) movement has emerged [10, 11] which is based on the construction and self-development of devices that can simulate a human torso or be useful for teaching resuscitation maneuvers, in order to promote mass training at low cost. Most DIY models we are aware of are either made with conventional materials, such as toilet paper, T-shirts or towels [10, 12] [13] or based on recycled material, especially plastic bottles [6, 14]. However, there is still a lack of knowledge on the usability of such manikins in the school. Studies on the acquisition of CPR skills via DIY manikin compared to commercial manikins are scarce. The development of DIY manikins must evolve and diversify to include models that are suitable for different contexts, learning outcomes and target groups. In a pedagogically ideal case, a DIY manikin may be able to replace with ingenuity those elements that have been shown to be effective in teaching resuscitation - such as feedback [15, 16], motivation [17], cognitive involvement [7] or gamification [18]- and evaluate their effect on the acquisition of skills.

Traditionally, conventional and unconventional manikins have focused on achieving the proper depth or rate of compressions, but it is well known that a key factor in resuscitation is its continuity, i.e., avoiding interruption of chest compressions (CC)[19], especially when performing the hands-only CPR (HO-CPR) technique[20]. However, to our knowledge there is no low-cost manikin for lay people that helps to train and understand this physiological concept.

Therefore, this study aims to evaluate the learning of CC skills by practicing with a Low Cost Manikin (LoCoMan) with visual qualitative feedback for continuous CC and to compare the results with the skills acquired by practice on a conventional manikin.

**MATERIAL AND METHOD**

**Participants**

A convenience sample of 193 schoolchildren from Spain participated in this research, aged between 10 and 12 years, corresponding to two academic years (5th and 6th year of primary school). The data broken down by groups and sex are shown in *table 1*. Exclusion criteria were any physical or mental impairments and any previous experience (theoretical or practical) in cardiopulmonary resuscitation.

The research was approved by the Ethics Committee of the Faculty of Education and Sport Sciences – University of Vigo (Spain) that supported (Code:03-250322). The study was authorized by the educational department of the participating schools and the parents (via written informed consent).

<< TABLE 1 >>

**Study Design**

A quasi-experimental, and cross-sectional study was carried out using two non-randomized intervention groups; the LoCoMan group (LG) trained with a hand-made Low-Cost Manikin and a control group (CG) practiced with a conventional pediatric manikin model, Resusci Junior (Laerdal, Norway). (Figure 1)

<< FIGURE 1 >>

**LoCoMan. Educational Project principles**

This project was based on the principles of significant, transversal and integrative learning[21, 22], focused on understanding the process by which continuous chest compressions achieve continuous circulation and cerebral perfusion. The LG built their manikins during one academic week in the physical education subject (2 sessions), in a coordinated manner with the subject of science (2 sessions), during which cardiocirculatory functions were learned (Figure 2). Children in the CG received the same science lesson, but without a comprehensive approach (no connection between contents).

<< FIGURE 2 >>

- Science subject: Participants were introduced to the cardiocirculatory system and the effect of HO-CPR on circulation. At the same time, they studied the functions of blood and the beating of the heart, the cardiac cycle, the transport of blood and the need for oxygen in different organs such as the brain.
- Physical Education subject: Participants built their manikin under guidance from a teacher. In small groups, they helped each other in the different phases of the build, which provoked collaboration and reflection. The materials and instructions for the build of LoCoMan are described in *figure 3;* more detailed instructions are available in an online supplementary video and assembly manual.
- Homework: In a first step before building LoCoMan, inspired by the flipped or inverted classroom theory [23, 24], the schoolchildren introduced the project to their families, and together they had to get some of the materials, such as cardboard to draw the human silhouette. In this way, the students began to integrate human anatomy, cardiac physiology and cardiopulmonary resuscitation. In a second step, at the end of the learning unit, schoolchildren had to take LoCoMan to their homes and teach HO-CPR to their relatives.

**LoCoMan's feedback mechanism** (Figure 3)

LoCoMan's blood circuit is made up of transparent plastic tubes, into which water (and some bubbles) had previously been introduced with a simple syringe. Once the “circulation tubes” are sealed, the water and numerous air bubbles are visible. This visually simulates the transport of oxygen by the blood. Visual feedback is based on visualizing the flow of fluid that is percussed (simulating the volume of systolic ejection) with each CC. The movement of the fluid, its circulation and the irrigation of the brain is easily perceptible during the maneuvers, as well as the immediate blood flow interruption when CC are interrupted. [see supplementary video]

<< FIGURE 3 >>

**CPR training (LG and CG).**

CPR training was guided by a physical education teacher, who had previously been trained by accredited instructors [25]. During a 1 hour-long physical education class, the following phases were carried out:

1) An explanation of the chain of survival was provided, including the sequence: recognizing cardiac arrest, calling 112, performing HO-CPR, and following the demonstration of chest compressions: depth, rate and correct position of execution. In the LG, feedback was provided by the movement of air bubbles through the "blood circuit" during compression, which should not stop. In the CG, feedback was provided by a tablet connected to the Resusci Junior (Laerdal, Norway) through the APP QCPR Training for IOS (Laerdal, Norway). The students in this group were instructed how to interpret the feedback from the tablet regarding three indicators: depth, full chest recoil, and rhythm (rate).

2) Practical demonstration of how to perform CPR on the manikin (the same for LG and CG).

3) The students performed CC guided by the teacher (the same for LG and CG).

4) Schoolchildren performed HO-CPR to the rate of the “La Macarena” song[26] (the same for LG and CG)..

5) Schoolchildren performed HO-CPR without any kind of teacher feedback (the same for LG and CG).

**Assessment of skills and analysis variables**

HO-CPR performance was analyzed during a separate testing session after the last practice session. Each student performed an individual test of 2-minutes. The assessment was carried out using the Little Junior QCPR manikin (Laerdal, Norway) programmed under the European Resuscitation Council guidelines for resuscitation 2021 (ERC2021): The gold standard describes chest compression (CC) depth of 50 - 60 mm, full chest recoil and a CC rate between 100 and 120 compressions per minute. Using the QCPR instructor app (Laerdal, Norway), the following variables were obtained: depth, full chest recoil and CC rate, as well as the global value of the quality of HO-CPR (QHO-CPR) in percentage. This device is widely used in the scientific literature for the evaluation of skills during resuscitation in simulation studies [18, 27, 28].

Manikin, interface and variables can be seen in *figure 1*. The analysis variables were: a) Number of CC in 2 minutes, b) Mean rate (R) of CC per minute during 2 minutes, c) average depth of CC in mm (D). And as percentage variables: d) overall QHO-CPR, e) CC with adequate chest recoil, f) CC with adequate depth and g) CC with adequate rate.

The percentage of students who reached different percentages of QHO-CPR was analyzed for 4 intervals: 0-24%, 25-49%, 50-69% and ≥70%. A result equal to or greater than 70% has been established as the highest standard[29].

**Statistical analysis**

Based on our hypothesis, CG and LG were compared in each dependent variable. An additional round of analyses was conducted within each school year (5th graders and 6th graders). All analyses were performed with the IBM SPSS Statistics Software version 20 for Windows. To describe the quantitative variables, measures of central tendency (median) and dispersion (interquartile range, IQR) were used. After checking the normality of the distributions with the Kolmogorov-Smirnov test, group (CG vs LG) comparisons of the continuous variables were made with the Mann-Whitney U test (nonparametric test) or with the Student's T test (parametric test). In statistically significant comparisons, the effect size (ES) was calculated using Rosenthal's r test (nonparametric test) or Cohen's d test (parametric test). To define the ES, the following classification was used: < 0.2 Trivial; 0.2 - 0.5 Small; 0.5 - 0.8 Moderate; 0.8 - 1.3 Large; ≥ 1.3 Very large. For the description of the percentage variables, absolute frequencies and relative frequencies were used. For the comparison of the groups in the percentage variables, the Chi Square test was used. In statistically significant comparisons, the ES was calculated using Cramer's V test. To define the ES, the following classification was used: 0.1 - 0.3: Small; 0.3 - 0.5: Medium; ≥ 0.5: Large. In the case of multiple comparisons between the groups (Chi Square), a p-value of 0.012 (0.05/4) was used for the Bonferroni correction. A significance level of p = 0.05 was assigned for all other analyses.

**RESULTS**

*Table 2* shows the dependent variables. The median number of CC performed during 2 min by the LG was 196 [161-221] while in the CG it was 217 [195-235], p=0.001. In the disaggregated analysis of continuous variables (mean rate and mean depth) also present significant differences, Rate; LG 99 [87-109] vs. CG 109 [100-122], p < 0.001 y, Depth; LG 43 mm [38-49] vs. CG 49 mm [43-55], p < 0.001, in the latter case with a large effect size (0.70).

In the analysis of the percentage of quality, LG performed at 57%[18-84] vs CG 71%[42-86], p=0.04 with a small effect size (0.15). The chest recoil rate was; LG 100%[92-100] vs. CG 98%[74-100], p=0.01, the percentage of adequate depth was; LG 3%[0-47] vs CG 40%[5-82], p < 0.001 and the percentage of CC at an adequate rate was not significantly different between groups; LG 31%[13-64] vs. CG 38%[21-62], p=0.24.

<< TABLE 2 >>

In the analysis of the percentage of QHO-CPR in the 5 sectors [0-24%, 25-49%, 50-69% and ≥70% ], at a global level no significant differences are found in the group of children who reach the higest sector of ≥70% .

In the 5^th^ grade, the best percentages are obtained by the control group but the 6^th^ grade the values of the CG and the LG group are similar, without significant differences in any sector. (Figure 4)

<< FIGURE 4 >>

In the analysis by school grades (supplementary table online), in the cohort of 5^th^ grade of primary education, all the variables showed significant differences in favor of the CG except in CC with adequate chest recoil. In the global analysis of quality (QHO-CPR variable), the CG (71[72-89]) performed better than the LG (39[4-82] p < 0.02). Analyzing the data from the 6^th^ grade cohort of primary education, there were significant differences between groups in the percentage variables, but no significant differences in the QHO-CPR.

**DISCUSSION**

The aim of this study was evaluate the learning of CC skills by practicing with a DIY manikin for continuous CC and to compare the results with the skills acquired by practice on a conventional manikin. Our findings corroborate the hypothesis that LoCoMan may be a useful device for teaching and learning HO-CPR in schoolchildren from the age of 10 and upwards.

We decided to work with 10-year-old schoolchildren, since chest compressions can be learned at this age with adequate training [30]. However, neither the group trained with LoCoMan nor the group that used the conventional manikin reached the recommended depth of compression (50-60mm) [31]. These findings may be related to the relatively low body mass at this age: the relationship of body mass and achieved depth of compressions is a limiting factor for children’s success at learning this skill [32, 33]. It should also be noted that the LoCoMan model cannot be compressed to the full depth of 50 mm (maximal depth is 35mm). This is a probable explanation for the finding that the control group obtained better results in depth variable in this study. Part of the purpose of building LoCoMan was to provide feedback on the continuity of compressions for continuous cerebral perfusion, a fundamental concept in HO-CPR. Based on the results obtained from number of CC, rate and mean rate percentage, we were able to verify that there was no interruption, neither in the control group using electronic feedback, nor in the LoCoMan group.

In the cohort analysis (i.e., separated by school year), LoCoMan led to better CPR quality in 6th graders (aged 11 and 12). Indeed, they performed practically equal to the control group, approaching the gold standard of 70% [29]. In other CPR variables such as full chest recoil or CC rate, significant variations were found between the two models, but these differences would not have clinical relevance because their values are close or very close to the gold standard of the resuscitation guidelines [34] (i.e. rate with LoCoMan was 104 CC/min vs. Laerdal Manikin 118 CC/min, gold standard 100-120 CC/min).

Hand-made manikins may contribute to the improvement of individual CPR and can make mass collective teaching possible, especially in low-resource settings [6] when there is no industry or government support. We consider that this project may be relevant because it could help promote the teaching of basic life support in schools or in informal contexts[6–10, 14, 35], which might have a significant impact on bystanders who perform CPR[4], for example by increasing confidence and readiness to act in an emergency [17, 36].

A relevant feature of LoCoMan is the qualitative feedback that allows the schoolchild to observe how the simulated blood circuit flows during chest compressions. To our knowledge, this is the only model that has visual feedback and integrates knowledge of circulatory physiology. Feedback during practice has been associated with better CPR quality and more effective learning [15], helping students achieve mastery of skills and shortening demonstration time [10]. Another noteworthy aspect is that throughout the training process, no manikin suffered significant deterioration, so this pilot tool is potentially sustainable for training or learning and although it is not commercialized, it can be locally manufactured since its components are readily available.

Most OHCAs occur at home (26). One additional aim of this educational project was to enable families to practice CPR at their homes. For this reason, the intervention included several “at-home steps”: Before building their manikin, learners involved their care-takers and relatives in the collection of recyclable materials to bring to school, and after the manikin was finished, the schoolchildren had to teach the skills acquired to their relatives. This process was not analysed as part of this paper, but it is also a mission of the project that should be evaluated in the future.

The presented project conforms to the main principles of KIDs SAVE LIVES: the dissemination of knowledge and training, encouraging fun and excitement during CPR (3). From an educational perspective, learning is stronger and more durable when it is based on connection of content, rather than analytical, isolated or mechanical learning. New learning methodologies and materials emerge and promoting learning-by-doing (5). LoCoMan is a project that combines the subject of science (knowledge of the human body) and physical education (first aid), all the while keeping the learner at the centre, as the protagonist promoting their own learning progress [21, 22].

This work has important practical implications: it shows that it is feasible and possible to build a low-cost manikin (about €5 in the European Region) and to integrate it into an integrative educational project, and outlines how this could be done. As a general reference, the cost of 100 LoCoMan units is less than 2 commercial manikins with feedback (i.e. as used in this study), and the learning outcomes are comparable. These and other evidence-based alternatives to expensive manikin training may be an incentive for teachers to attempt teaching CPR, but also for education outside the formal environment.

*Study limitations*

We acknowledge that this study has a few limitations. The sample was located in a specific region, so it is not representative of all children worldwide. As the classes were grouped by academic years, there was a slight age overlap around the age of 11 years. A distribution by biological age (10, 11 and 12 years) could be more appropriate, but due to the educational dynamics of the school system, this has not been possible. Another factor that is necessary to note is that the LoCoMan is part of a multidisciplinary educational project, and this circumstance might improve the motivation of schoolchildren and relatives to perform well. A relevant and limiting aspect of the study is that a portion of the learning in the LoCoMan group may possibly be influenced by the overall project rather than exclusive training in HO-CPR. This methodological limitation also serves as a strength of this project, which extends beyond the building of a manikin. Another limitation is that, as each student made their own LoCoMan there may have been small variations between manikins as it is not a standardized model. The LoCoMan structure allows a maximum compression depth of 35 mm (based on measurements with a CPR meter (Laerdal, Norway, seesupplementary video online) which may be the most plausible justification for achieving 3% of compressions at the indicated depth compared to the 40% achieved by the children who trained on the commercial manikin. Future versions of LoCoMan will address this issue.

**CONCLUSION**

The use of a low-cost, hand-made manikin with visual feedback might be an alternative for training and learning CPR in schools, especially when commercial manikins are not available. Low-cost manikins can be integrated into an educational project that promotes learning of CPR

**FUNDING**

Research funded by Instituto de Salud Carlos III (ISCIII) -PI20/01355- Co-funded by the European Union (EU)

Research funded by Instituto de Salud Carlos III (ISCIII) -PI23/00687- Co-funded by the European Union (EU).

**Conflict of Interest Disclosures:** None reported.

**Author Contributions:**

Martinez-Isasi S y Barcala-Furelos R had full access to all of the data in the study and take responsibility for the integrity of the data and the accuracy of the data analysis.

*Concept and design:* Peixoto-pino, Barcala-Furelos and ´ Rodriguez-Nuñez

*Acquisition, analysis, or interpretation of data: Peixoto-Pino, Barcala-Furelos, Otero-Agra, Martínez-Isasi and Rico-Díaz.*

*Drafting of the manuscript*: Barcala-Furelos and Van Duijn

*Critical revision of the manuscript for important intellectual content:* Rodriguez-Nuñez and Martínez-isasi

*Statistical analysis:* Otero-Agra

*Obtained funding:* Martínez-isasi

*Administrative, technical, or material support: Peixoto-Pino; Barcala-Furelos and Martínez-isasi*

*Supervision:* Barcala-Furelos and Martínez-Isasi

***Ethics declarations***

Ethics approval

The study was performed in line with the principles of the Declaration of Helsinki and was approved by the Ethical Committee of the Faculty of Education and Sport Sciences – University of Vigo (Spain).

Consent to participate

Written informed consent was obtained from the parents and verbal consent was obtained from all participating children.

**REFERENCES**

1. Perkins GD, Gräsner J-T, Semeraro F, et al (2021) European Resuscitation Council Guidelines 2021: Executive summary. Resuscitation 161:1–60. https://doi.org/10.1016/j.resuscitation.2021.02.003

2. Nakahara S, Tomio J, Ichikawa M, et al (2015) Association of Bystander Interventions With Neurologically Intact Survival Among Patients With Bystander-Witnessed Out-of-Hospital Cardiac Arrest in Japan. JAMA 314:247–254. https://doi.org/10.1001/jama.2015.8068

3. Cheng A, Magid DJ, Auerbach M, et al (2020) Part 6: Resuscitation Education Science: 2020 American Heart Association Guidelines for Cardiopulmonary Resuscitation and Emergency Cardiovascular Care. Circulation 142:S551–S579. https://doi.org/10.1161/CIR.0000000000000903

4. Greif R, Lockey AS, Conaghan P, et al (2015) European Resuscitation Council Guidelines for Resuscitation 2015: Section 10. Education and implementation of resuscitation. Resuscitation 95:288–301. https://doi.org/10.1016/j.resuscitation.2015.07.032

5. Böttiger BW, Van Aken H (2015) Kids save lives--Training school children in cardiopulmonary resuscitation worldwide is now endorsed by the World Health Organization (WHO). Resuscitation 94:A5-7. https://doi.org/10.1016/j.resuscitation.2015.07.005

6. Nakagawa NK, Oliveira KMG, Lockey A, et al (2021) Effectiveness of the 40-Minute Handmade Manikin Program to Teach Hands-on Cardiopulmonary Resuscitation at School Communities. Am J Cardiol 139:126–130. https://doi.org/10.1016/j.amjcard.2020.09.032

7. Rabanales-Sotos J, Guisado-Requena IM, Leiton-Espinoza ZE, et al (2022) Development and Validation of a Novel Ultra-Compact and Cost-Effective Device for Basic Hands-On CPR Training: A Randomized, Sham-Controlled, Blinded Trial. Int J Environ Res Public Health 19:15228. https://doi.org/10.3390/ijerph192215228

8. Barry M, Dixon M, Armstrong C, Keane F (2019) The Pillow Project, Infant Choking, and Basic Life Support Training for Prospective Parents: A Low-Cost Intervention for Widespread Application. J Perinat Neonatal Nurs 33:260–267. https://doi.org/10.1097/JPN.0000000000000397

9. Anuntaseree S, Kalkornsurapranee E, Yuenyongviwat V (2019) Efficacy of and Satisfaction with an In-house Developed Natural Rubber Cardiopulmonary Resuscitation Manikin. West J Emerg Med 21:91–95. https://doi.org/10.5811/westjem.2019.10.43004

10. Ohle R, Moskalyk M, Boissonneault E, et al (2021) Is a homemade cardiopulmonary resuscitation (CPR) trainer non-inferior to a commercially available CPR mannequin in teaching high-quality CPR? A non-inferiority randomized control trial. Resusc Plus 6:100134. https://doi.org/10.1016/j.resplu.2021.100134

11. van Duijn T, Tobler E, Abaecherli R (2022) Evidence-base and feasibility of using homemade manikins to teach and learn resuscitation skills. Australasian Skill Acquisition Network (ASAN) annual conference, Melbourne, Australia. In: Australasian Skill Acquisition Network (ASAN) annual conference. Melbourne, Australia.

12. Wanner GK, Osborne A, Greene CH (2016) Brief compression-only cardiopulmonary resuscitation training video and simulation with homemade mannequin improves CPR skills. BMC Emerg Med 16:45. https://doi.org/10.1186/s12873-016-0110-5

13. Nehra A, Ravindra P, Bhat R, et al (2024) Comparison between a low-cost model (CPR Pillow) and a mannequin in training hands only cardiopulmonary resuscitation (CPR): A randomised trial. Resusc Plus 17:100518. https://doi.org/10.1016/j.resplu.2023.100518

14. Birkun A (2021) Do-it-yourself PET-bottle manikin: An understudied means for enhancing massive CPR training in a simple and affordable way. Afr J Emerg Med Rev Afr Med Urgence 11:309–310. https://doi.org/10.1016/j.afjem.2021.04.004

15. Eshel R, Wacht O, Schwartz D (2019) Real-Time Audiovisual Feedback Training Improves Cardiopulmonary Resuscitation Performance: A Controlled Study. Simul Healthc J Soc Simul Healthc 14:359–365. https://doi.org/10.1097/SIH.0000000000000390

16. Abelairas-Gómez C, Rodríguez-Núñez A, Vilas-Pintos E, et al (2015) Effects of real-time audiovisual feedback on secondary-school students’ performance of chest compressions. Emergencias 27:189–192

17. Weidenauer D, Hamp T, Schriefl C, et al (2018) The impact of cardiopulmonary resuscitation (CPR) manikin chest stiffness on motivation and CPR performance measures in children undergoing CPR training-A prospective, randomized, single-blind, controlled trial. PloS One 13:e0202430. https://doi.org/10.1371/journal.pone.0202430

18. Otero-Agra M, Barcala-Furelos R, Besada-Saavedra I, et al (2019) Let the kids play: gamification as a CPR training methodology in secondary school students. A quasi-experimental manikin simulation study. Emerg Med J 36:653–659. https://doi.org/10.1136/emermed-2018-208108

19. Cunningham LM, Mattu A, O’Connor RE, Brady WJ (2012) Cardiopulmonary resuscitation for cardiac arrest: the importance of uninterrupted chest compressions in cardiac arrest resuscitation. Am J Emerg Med 30:1630–1638. https://doi.org/10.1016/j.ajem.2012.02.015

20. Kern KB, Timerman S, Gonzalez MM, Ramires JA (2011) Optimized approach in cardiocerebral resuscitation. Arq Bras Cardiol 96:e77-80. https://doi.org/10.1590/s0066-782x2011000400020

21. Agra G, Formiga NS, Oliveira PS de, et al (2019) Analysis of the concept of Meaningful Learning in light of the Ausubel’s Theory. Rev Bras Enferm 72:248–255. https://doi.org/10.1590/0034-7167-2017-0691

22. Barber JP (2012) Integration of Learning: A Grounded Theory Analysis of College Students’ Learning. Am Educ Res J 49:590–617. https://doi.org/10.3102/0002831212437854

23. Akçayır G, Akçayır M (2018) The flipped classroom: A review of its advantages and challenges. Comput Educ 126:334–345. https://doi.org/10.1016/j.compedu.2018.07.021

24. Bergmann J, Sams A (2012) Flip Your Classroom: Reach Every Student in Every Class Every Day. International Society for Technology in Education

25. Pichel López M, Martínez-Isasi S, Barcala-Furelos R, et al (2018) Un primer paso en la enseñanza del soporte vital básico en las escuelas: la formación de los profesores. An Pediatría 89:265–271. https://doi.org/10.1016/j.anpedi.2017.11.002

26. Oulego-Erroz I, Busto-Cuiñas M, García-Sánchez N, et al (2011) A popular song improves CPR compression rate and skill retention by schoolchildren: A manikin trial. Resuscitation 82:499–500. https://doi.org/10.1016/j.resuscitation.2011.01.001

27. Arrogante O, González-Romero GM, Caperos JM, et al (2020) Quality of cardiopulmonary resuscitation: Degree of agreement between instructor and a feedback device during a simulation exercise. Int Emerg Nurs 53:100907. https://doi.org/10.1016/j.ienj.2020.100907

28. Otero-Agra M, Jorge-Soto C, Cosido-Cobos ÓJ, et al (2022) Can a voice assistant help bystanders save lives? A feasibility pilot study chatbot in beta version to assist OHCA bystanders. Am J Emerg Med 61:169–174. https://doi.org/10.1016/j.ajem.2022.09.013

29. Perkins GD, Colquhoun M, Simons R (2004) ABC of resuscitation, 5th ed. BMJ books, London

30. Bohn A, Van Aken HK, Möllhoff T, et al (2012) Teaching resuscitation in schools: annual tuition by trained teachers is effective starting at age 10. A four-year prospective cohort study. Resuscitation 83:619–625. https://doi.org/10.1016/j.resuscitation.2012.01.020

31. Olasveengen TM, Semeraro F, Ristagno G, et al (2021) European Resuscitation Council Guidelines 2021: Basic Life Support. Resuscitation 161:98–114. https://doi.org/10.1016/j.resuscitation.2021.02.009

32. Abelairas-Gómez C, Rodríguez-Núñez A, Casillas-Cabana M, et al (2014) Schoolchildren as life savers: at what age do they become strong enough? Resuscitation 85:814–819. https://doi.org/10.1016/j.resuscitation.2014.03.001

33. Jones I, Whitfield R, Colquhoun M, et al (2007) At what age can schoolchildren provide effective chest compressions? An observational study from the Heartstart UK schools training programme. BMJ 334:1201. https://doi.org/10.1136/bmj.39167.459028.DE

34. Monsieurs KG, Nolan JP, Bossaert LL, et al (2015) European Resuscitation Council Guidelines for Resuscitation 2015: Section 1. Executive summary. Resuscitation 95:1–80. https://doi.org/10.1016/j.resuscitation.2015.07.038

35. Sá-Couto C, Ferreira AM, Almeida D, et al (2018) Evaluation of skills acquisition using a new low-cost tool for CPR self-training. Porto Biomed J 3:e8. https://doi.org/10.1016/j.pbj.0000000000000008

36. Farquharson B, Dixon D, Williams B, et al (2023) The psychological and behavioural factors associated with laypeople initiating CPR for out-of-hospital cardiac arrest: a systematic review. BMC Cardiovasc Disord 23:19. https://doi.org/10.1186/s12872-022-02904-2

37. Gräsner J-T, Lefering R, Koster RW, et al (2016) EuReCa ONE⿿27 Nations, ONE Europe, ONE Registry: A prospective one month analysis of out-of-hospital cardiac arrest outcomes in 27 countries in Europe. Resuscitation 105:188–195. https://doi.org/10.1016/j.resuscitation.2016.06.004

Table 1. Characteristics of the sample.

| Variables | | Total  n = 193 | | LoCoMan group  n = 91 | | Control group  n = 102 | | *p* value |
| --- | --- | --- | --- | --- | --- | --- | --- | --- |
| Academic year | | n | (%) | n | (%) | n | (%) |  |
|  | 5ºPE [10-11 year] | 106 | (55 %) | 45 | (50 %) | 61 | (60 %) | p = 0.15 |
|  | 6ºPE [11-12 year] | 87 | (45 %) | 46 | (51 %) | 41 | (40 %) |  |
| Sex | | n | (%) | n | (%) | n | (%) |  |
|  | Female | 106 | (55 %) | 56 | (61 %) | 50 | (49 %) | p = 0.08 |
|  | Male | 87 | (45 %) | 35 | (39 %) | 52 | (51 %) |  |
| PE (Primary Education) | | | | | | | | |

| Table 2: CPR variables | | | | | |
| --- | --- | --- | --- | --- | --- |
| variable | LoCoMan group  No. = 91 | | Control group  No. = 102 | | *p* values (p) and  Efect Size (ES) |
|  | Median | IQR | Median | IQR |  |
| Number of CC | 196 | (161 - 221) | 217 | (195 - 235) | p = 0.001 (ES 0.26) |
| Mean rate (CC/min) | 99 | (87 - 109) | 109 | (100 - 122) | p < 0.001 (ES 0.34) |
| Mean depth (mm) * | 43 | (38 - 49) | 49 | (43 - 55) | p < 0.001  (ES 0.70) |
| QHO-CPR (%) | 57 | (18 - 84) | 71 | (42 - 86) | p = 0.04 (ES 0.15) |
| CC with full chest recoil (%) | 100 | (92 - 100) | 98 | (74 - 100) | p = 0.01 (ES 0.18) |
| CC with adequate depth (%) | 3 | (0 - 47) | 40 | (5 - 82) | p < 0.001 (ES 0.33) |
| CC with adequate rate (%) | 31 | (13 - 64) | 38 | (21 - 62) | p = 0.30 |
| IQR: Interquartile range; N: Absolute frequency; (%): Relative frequency.  For quantitative variables: Mann-Whitney's U test with Rosenthal's test for Effect Size  * For quantitative variables: Student's t test with Cohen's d test for Effect Size  For Effect Size classification: < 0.2: Trivial; 0.2 - 0.5: Small; 0.5 - 0.8: Moderate; 0.8 - 1.3: Large; > 1.3: Very Large  For qualitative variables: Chi Square test with Cramer's V test for Effect Size  For Effect Size (ES) classification: 0.1 - 0.3: Small; 0.3 - 0.5: Medium; ≥0.5:Large | | | | | |

Supplementary table 1

| Table 3: CPR variables by age | | | | | |
| --- | --- | --- | --- | --- | --- |
| 5 th PE [10 and 11 yo] | | | | | |
| variables | Locoman group (LG)  No. = 45 | | Control group (CG)  No. = 61 | | Significance |
|  | Median | IQR | Median | IQR |  |
| Number of CC | 189 | (138 - 204) | 207 | (190 - 223) | p = 0.001 (0.32) |
| Rate (CC/min) | 96 | (86 - 103) | 106 | (97 - 116) | p < 0.001 (0.36) |
| Depth (mm) | 41 | (37 - 48) | 49 | (42 - 53) | p < 0.001 (0.40) |
| CPR Quality (%) | 39 | (4 - 82) | 72 | (38 - 89) | p = 0.02 (0.24) |
| CC with adequate recoil (%) | 100 | (98 - 100) | 99 | (74 - 100) | p < 0.002 (0.30) |
| CC with adequate depth (%) | 2 | (0 - 36) | 40 | (5 - 77) | p < 0.001 (0.37) |
| CC with adequate rate (%) | 28 | (9 - 46) | 43 | (24 - 67) | p = 0.007 (0.26) |
| 6 th PE [11 and 12 yo] | | | | | |
| variables | Locoman group (LG)  No. = 46 | | Control group (CG)  No. = 41 | | Significance |
|  | Median | IQR | Median | IQR |  |
| NCC | 215 | (169 - 224) | 233 | (211 - 249) | p = 0.008 (0.29) |
| R (CC/min) * | 104 | (89 - 110) | 118 | (103 - 127) | p < 0.001 (0.84) |
| D (mm) | 46 | (40 - 51) | 49 | (44 - 57) | p = 0.011 (0.27) |
| QHO-CPR (%) | 68 | (31 - 88) | 71 | (54 - 81) | p = 0.66 |
| CC with full chest recoil (%) | 98 | (82 - 100) | 93 | (72 - 100) | p = 0.28 |
| CC with adequate depth (%) | 13 | (0 - 59) | 33 | (6 - 91) | p = 0.005 (0.30) |
| CC with adequate rate (%) | 42 | (18 - 80) | 32 | (13 - 55) | p = 0.23 |
| NCC: Number of CC. A: Mean rate (CC/min). D: Mean depth (mm), QHO-CPR: Overall quality of CPR in %. CC: Chest compression. PE. Primary school  IQR: Interquartile range; N: Absolute frequency; (%): Relative frequency.  For quantitative variables: Mann-Whitney's U test with Rosenthal's test for Effect Size  * For quantitative variables: Student's t test with Cohen's d test for Effect Size  For Effect Size classification: < 0.2: Trivial; 0.2 - 0.5: Small; 0.5 - 0.8: Moderate; 0.8 - 1.3: Large; > 1.3: Very Large  For Effect Size classification: 0.1 - 0.3: Small; 0.3 - 0.5: Medium; ≥0.5:Large | | | | | |

**Legends**

Figure 1. Flow chart design

Figure 2. Integrative Educational Project learn-by-doing LoCoMan

Figure 3. How to make a LoCoMan (step by step) and in a supplementary video online.

Figure 4. Quality CPR sectors and percentage of schoolchildren by group and grade.
